# Live Imaging-Based Model Selection Reveals Periodic Regulation of the Stochastic G1/S Phase Transition in Vertebrate Axial Development

**DOI:** 10.1371/journal.pcbi.1003957

**Published:** 2014-12-04

**Authors:** Mayu Sugiyama, Takashi Saitou, Hiroshi Kurokawa, Asako Sakaue-Sawano, Takeshi Imamura, Atsushi Miyawaki, Tadahiro Iimura

**Affiliations:** 1 Laboratory for Cell Function and Dynamics, Advanced Technology Development Group, Brain Science Institute, RIKEN, Wako-city, Saitama, Japan; 2 Department of Molecular Medicine for Pathogenesis, Graduate School of Medicine, Ehime University, Shitsukawa, Toon-city, Ehime, Japan; 3 Division of Bio-Imaging, Proteo-Science Center (PROS), Ehime University, Shitsukawa, Toon-city, Ehime, Japan; 4 Translational Research Center, Ehime University Hospital, Shitsukawa, Toon-city, Ehime, Japan; New York Medical College, United States of America

## Abstract

In multicellular organism development, a stochastic cellular response is observed, even when a population of cells is exposed to the same environmental conditions. Retrieving the spatiotemporal regulatory mode hidden in the heterogeneous cellular behavior is a challenging task. The G1/S transition observed in cell cycle progression is a highly stochastic process. By taking advantage of a fluorescence cell cycle indicator, Fucci technology, we aimed to unveil a hidden regulatory mode of cell cycle progression in developing zebrafish. Fluorescence live imaging of Cecyil, a zebrafish line genetically expressing Fucci, demonstrated that newly formed notochordal cells from the posterior tip of the embryonic mesoderm exhibited the red (G1) fluorescence signal in the developing notochord. Prior to their initial vacuolation, these cells showed a fluorescence color switch from red to green, indicating G1/S transitions. This G1/S transition did not occur in a synchronous manner, but rather exhibited a stochastic process, since a mixed population of red and green cells was always inserted between newly formed red (G1) notochordal cells and vacuolating green cells. We termed this mixed population of notochordal cells, the G1/S transition window. We first performed quantitative analyses of live imaging data and a numerical estimation of the probability of the G1/S transition, which demonstrated the existence of a posteriorly traveling regulatory wave of the G1/S transition window. To obtain a better understanding of this regulatory mode, we constructed a mathematical model and performed a model selection by comparing the results obtained from the models with those from the experimental data. Our analyses demonstrated that the stochastic G1/S transition window in the notochord travels posteriorly in a periodic fashion, with doubled the periodicity of the neighboring paraxial mesoderm segmentation. This approach may have implications for the characterization of the pathophysiological tissue growth mode.

## Author Summary

Cell cycle progression is considered to involve a cellular time-counting machinery for proper morphogenesis and patterning of tissues. Therefore, it is important to understand the regulatory mode of cell cycle progression during physiological and pathological tissue growth, which will benefit tissue engineering therapy and tumor diagnosis. Since cell cycle progression is a highly variable process, it is a challenging task to retrieve the spatiotemporal regulatory mode hidden in heterogeneous cellular behavior. To overcome this issue, we took advantage of live imaging analyses with a fluorescence cell cycle indicator, Fucci technology, and mathematical modeling of developing zebrafish fish embryo as a model system of growing tissue. Our result demonstrated that the developmental growth of embryonic axis progressed in a rhythmic fashion. The presented analyses will benefit the characterization of the regulatory mode of tissue growth, in both physiological and pathological development, such as that involving tumor formation, thus may contribute to cancer diagnosis.

## Introduction

The development of multicellular organisms is a highly coordinated process, in which cell proliferation and sequential changes in cellular identities are spatiotemporally regulated, through which patterned tissues and organs are ultimately formed. As a system to ensure the precision and reproducibility of the developmental process, the concept of “intrinsic time” has been postulated [1], [2]. Cell cycle progression has long been considered to involve a cellular time-counting machinery for proper morphogenesis and patterning of tissues. This notion is fundamentally supported by observations of increased mitotic activity in populations of cells that transiently appear during the developmental process. The presence of temporal waves of mitotic activity in the developing limb mesenchyme is reported to correlate with a segmented skeletal pattern, thus possibly accommodating the positioning of bones and joints in limbs [3]. In addition, a clustered mitotic activity observed in the endoderm are proposed to be responsible for morphogenetic folding to form the digestive tract [4]. Furthermore, periodic surges of mitotic activity in the paraxial mesoderm have been repeatedly observed in concert with reiterate somite formation in embryonic tissue [5]–[7]. Since somites principally endow a segmented architecture to the axial skeleton and its associated muscles and neurons, timed machineries of somite formation provide a fundamental system for body plan and anatomical structure [8]–[10]. The periodic formation of somites is regulated by the segmentation clock, which exhibits an oscillatory expression of signaling molecules related to Notch, Wnt and Fgf [9], [11]–[14]. Though it has been proposed that cell cycle progression regulates periodic somite formation, as described above, the current findings argue against the idea that the cell cycle clock is involved as an oscillator of the segmentation clock [15]–[17].

It is well established that cell cycle progression is a highly variable process. A phenomenological description of the stochastic cell cycle progression has been reported [18]–[22], and mathematical models that account for the variable transition timing in cell cycle progression have been proposed [19], [23]–[30]. Based on *in vitro* observations of mammalian cell cultures, a conceptual framework of “the restriction point” of the G1/S transition has been proposed [22]. The restriction point divides the G1 phase into the G1-postmitosis phase (G1-pm) and the G1-pre S phase (G1-ps), in which cells are able to proliferate dependent and independent of mitotic stimuli, respectively. G1-pm is highly constant in time length (approximately three hours), while the duration of G1-ps varies considerably. The restriction point is currently understood to extend the timing of phosphorylation of Rb proteins by Cyclin D1, thus releasing E2F in order to initiate S phase entry. Mathematical modeling analyses have also suggested a bistable mechanism to control the restriction point in the mammalian G1/S transition [31]–[35]. Yao et al., experimentally demonstrated bistable E2F activation that directly correlated with the ability of a cell to traverse the restriction point by temporally monitoring the E2F transcriptional activity with stimuli of various magnitudes, thus validating that the RB-E2F pathway involving multiple positive feedback loops can generate bistability; namely, by forming the Rb-E2F bistable switch [36]. This Rb-E2F bistable switch is further extended to work even when subjected to noise, which supported the proposed models to account for the temporal variability in the G1-S transition [37]. In this stochastic model, both cellular intrinsic and extrinsic noise can be taken into account. The intrinsic noise results from the stochastic nature of biochemical interactions due to the stochastic gene expression levels in each single cell, while the extrinsic noise arises from heterogeneous properties of a cell, such as the cell's size, shape, cell cycle phase and cell division [38]–[43].

**Figure 1:**
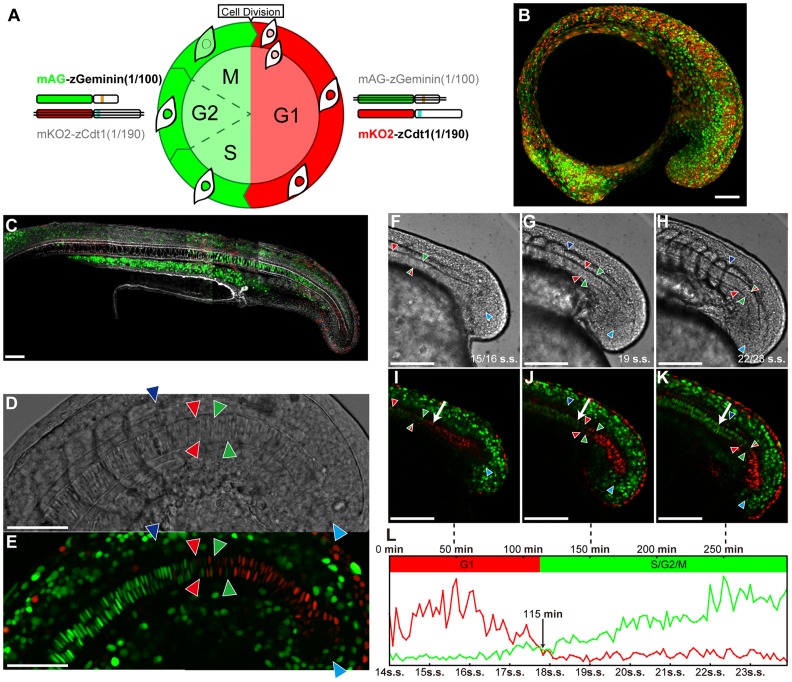
Progressive mode of the stochastic G1/S transition in the notochordal cells during embryonic body elongation. (A) Fucci (fluorescent ubiquitination-based cell cycle indicator) labels nuclei of G1 phase cells red (orange) and those of cells in the S/G2/M phase green. During the cell cycle of cells expressing Fucci, two chimeric proteins, mKO2-zCdt1 and mAG-zGeminin, reciprocally accumulate in the G1 and S/G2/M phases [48], [49] (see [48] for detailed molecular construction). (B) Cecyil (cell cycle illuminated: a zebrafish line producing zFucci) embryos at the 17 somite stage. A Z-stacking lateral view of optical sections. The anterior view is to the left, the posterior is to the right. (C) A mid-sagittal optical section of a fixed Cecyil embryo at 22 hpf counterstained with phalloidin Alexa Fluor 647. (D, E) Mid-sagittal optical section of a fixed Cecyil embryo at 18 hpf. A DIC (differential integrative phase contrast) image (D) and fluorescence image (E) are shown. The posterior-most green cell (PGC) and anterior-most red cell (ARC) in the upper and lower notochordal cells are indicated by green and red arrowheads, respectively. The posterior tip of the precursor pool of the notochord and the last formed somitic border are marked by sky blue and blue arrowheads, respectively. (F–K) Mid-sagittal optical sections of a developing Cecyil embryo. DIC images obtained at three different time points (F–H) and their corresponding fluorescence images are shown (I–K). The colored arrowheads indicate the same structures of cells as shown in D and E. The white arrow indicates a single cell whose cell cycle phase was transitioning from G1 (I) to S (J and K). Embryonic stages are indicated by somite stages (s.s.). (L) The temporal changes in the red and green fluorescence intensity of the cell indicated by the white arrow in G–H are plotted. The time course of the live imaging observations and embryonic stages is indicated in minutes and somite stages (s.s.), respectively. The vertical broken lines indicate the observation times corresponding to the images in g–i. The crossing time point of the red and green lines is indicated by the yellow point (115 min, in 18 somite stages). This time was selected as the time at which the G1/S transition occurred (see Methods). White scale bars: 100 *µm*. Anterior to the left, posterior to the right. Dorsal to the top, ventral to the bottom. s.s, somite stage.

Generally, during *in vivo* tissue development, biochemical phenomena are intrinsically associated with stochasticity, in which fluctuations in cellular responses are observed in populations of cells exposed to the same environmental conditions [38], [44]–[47]. In multicellular organism development, heterogeneous cellular behavior, such as the G1/S transition, possibly blinds regulatory events. Therefore, it is relevant to develop novel approaches to numerically estimate noise strength or the probability of a stochastic cellular response in multicellular organisms, which would unveil a regulatory mode of tissue growth associated with morphogenesis and patterning. Our research group previously reported the findings of whole-view imaging of cell cycle progression during embryonic development obtained using fluorescence live imaging of Cecyil, a zebrafish line that genetically carries a fluorescence cell cycle indicator, Fucci [48]. The Fucci system applies ubiquitin-based cell cycle phase-specific oscillators that are fused to distinct colored fluorescence proteins [49], which demarcates cell nuclei in the G1 and S/G2/M phases in red and green, respectively (Figures 1A, B). By taking advantage of the Fucci system, this study aimed to establish a method to clarify the regulatory mode of cell cycle progression veiled in the stochastic behavior of developing tissue. Quantitative analyses of our live imaging data combined with mathematical modeling demonstrated that the stochastic G1/S transition window in the notochord travels posteriorly in a periodic fashion with doubled periodicity of neighboring paraxial mesoderm segmentation. The results of our analyses are expected to benefit the characterization of the regulatory mode of tissue growth, in both physiological and pathological development, such as that involving tumor formation.

## Results

### Cecyil revealed that the G1/S transition in notochordal cells is associated with progressive somite segmentation and body axis elongation

Mid-sagittal optical sections of fixed Cecyil embryos counterstained with phalloidin (Figure 1C) and live observation of fluorescence and DIC (differential interference contrast) images (Figure 1 D to K, Movie S1) demonstrated that morphological differentiation of notochordal cells is associated with cell cycle progression. Newly formed notochordal cells in the posterior region expressed intense red fluorescence signals in their nuclei, being arranged to form two arrays of red cells. These cells arose from their precursor pools of chord neural hinges that comprised a mixture populations of dark-red cells and green cells (indicated by the sky blue arrowheads in Figure 1 D to K), suggesting that G1 entry is tightly related to initial notochord formation. The second color switches of the Fucci signals (G1 red to S/G2/M green, indicated by the red and green arrowheads in Figure 1 D to K) were observed prior to the initiation of cell vacuolization (Figure 1C); therefore, this initial G1/S transition is possibly required for notochord differentiation. In most of the observations, we observed a mixed population of green and red cells between the anterior green cells and posterior red cells, possibly suggesting the stochastic behavior of the G1/S transition in each cell. Therefore, we termed this mixed population of green and red cells the G1/S transition window. As embryonic development progressed, the G1/S transition window moved posteriorly by following the direction of body axis elongation (Figure 1 F to K). Comparing the DIC and fluorescence images, we mapped the position of a newly formed somite (indicated by the blue arrowheads in Figure 1 F to K) and that of the G1/S transition window (red and green arrowheads). This observation indicated that the initial G1/S transition is always observed at a position posterior to newly formed somites (Figure 1D). These findings suggest that the regulatory mode of the G1/S transition in notochordal cells is related to progressive modes of embryonic body axis elongation and somite segmentation.

### Scoring the mode of G1/S transition in notochordal cells

In order to quantitatively analyze the mode of G1/S transition in notochordal cells, we digitalized two cellular states, the G1 and S/G2/M phase, using image processing. We initially tracked each single notochordal cell by measuring the fluorescence intensity of the red and green signals in live imaging data. Figure 1J shows the representative tracking data of a single cell indicated by the white arrows in Figure 1 G to H. The time point of the G1/S transition was determined as the crossing point of the red and green lines of the fluorescence intensity, which occurred at 115 minutes in Figure 1J. For details, please see the Methods section.

**Figure 2:**
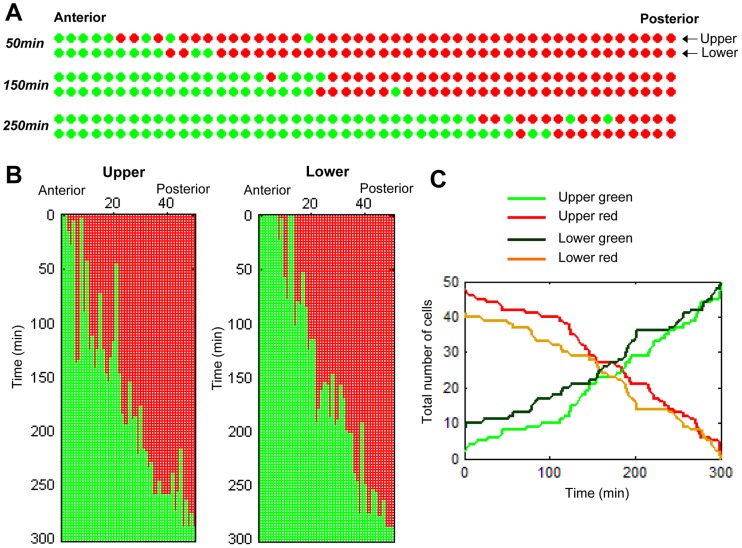
Scoring of the mode of the G1/S transition in the notochordal cells. (A) Binarized images of notochordal cells at three different time points (*t = 50 min*, *150 min and 250 min*). Cells in the G1 and S phases are indicated by red and green points, respectively. (B) The dynamics of cell cycle progression are represented by a two-dimensional map on the plane of time and space (the anterior-posterior axis). The upper and lower sequences along the anterior-posterior axis are drawn individually. (C) Total number of red (G1) and green (S) cells as a function of time. The green and red lines, and the dark-green and orange lines denote the total number of S and G1 cells of the upper and lower sequences, respectively.

Based on this method, we next plotted the binary valued cellular states of the upper (dorsal) and lower (ventral) columns of cells in a single dimension of the anterior to posterior position (Figure 2A, Movie S2). This plotting was then applied to succeeding time lapse images. Therefore, the second dimension was introduced in order to demonstrate the temporal dynamics of the G1/S transition (Figure 2B). Notably, the spatial positions of the G1/S transition were observed to overtly progress posteriorly, although the transition in each cell did not occur in the exact order of their position, but rather in a stochastic fashion. When the total number of green and red cells was scored at each time point, it was obvious that the stochastic G1/S transition progressed posteriorly as time progressed (Figure 2C), although the modes of the upper and lower cells were not entirely synchronized.

**Figure 3:**
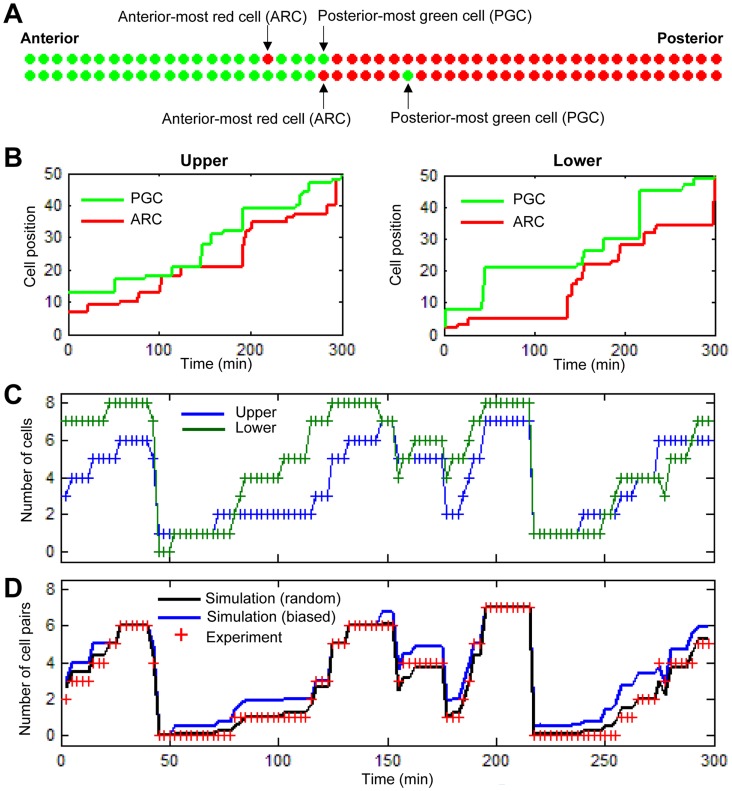
Systematic analyses of binarized images of the G1/S transition. (A) Two quantitative indices, the anterior-most red cell (ARC) and posterior-most green cell (PGC), are introduced as illustrated. (B) The positions of the ARC and PGC as a function of time. The upper and lower sequences of notochordal cells along the anterior-posterior axis are drawn individually. (C) The total number of green (S) cells in the G1/S transition window, defined as seven cells anterior to the PGC, as well as the PGC (total: 8 cells) (also demonstrated in Figure S1), as a function of time. The blue and green lines with ‘+’ markers indicate the upper and lower sequence data, respectively. (D) Number of green cell pairs in the G1/S transition window as a function of time. The red ‘+’ markers indicate the data obtained from the experiments. The black and blue lines indicate data obtained using random and biased simulation, respectively (see Methods).

In order to further investigate the mode of this stochastic G1/S transition in more detail, we focused on spatiotemporal changes in a mixed population of red and green cells that demarcated the stochastic G1/S transition window. We scored the positions of the anterior-most red cell (ARC) and posterior-most green cell (PGC), which demarcate the anterior and posterior positions of the stochastic G1/S transition window, respectively, at each time point of observation (Figure 3A, Figure S1). Scoring of the position of the ARC and PGC showed a pattern of step-wise progression in which the position of the ARC (indicated by the red lines in Figure 3B and Figures S2A and S3A) followed and occasionally caught up to that of the PGC (green lines in Figure 3B and Figures S2A and S3A). Therefore, the stochastic G1/S transition window appeared to repeatedly widen and shorten its width (see the space enclosed by the green and red lines in the graphs shown in Figure 3B and Figures S2A and S3A).

We applied another method of scoring in order to further analyze the spatiotemporal dynamics of the G1/S transition window, in which the total number of green cells in seven cells anterior to the PGC, including the PGC itself, (total: 8 cells) at each time point was scored (Figure S1). Interestingly, this scoring system demonstrated that the number of green cells in this defined area synchronously oscillated in the upper and lower columns over a period of 60 minutes or more (Figure 3C, Figure S2B and S3B).

### S phase entry in notochordal cells is independent of neighboring cell states

We next investigated whether the state of the cell cycle phase affects the G1/S transition of neighboring cells. The total number of green cell pairs (the upper and lower cells at the same position are both labeled in green) in seven pairs anterior to the PGC and a pair of cells including the PGC was scored (Figure 3D, red ‘+’ markers). Consistent with the data presented in Figure 3C, the temporal changes in the number of green cell pairs also exhibited an oscillatory behavior. We next compared these data with the random or biased distribution patterns generated by *in silico* simulation (see the Methods section). The expectation values of the number of green cell pairs for the random and biased cases are shown in Figure 3D in black and blue lines, respectively. The experimental data shown by the red ‘+’ markers exhibited a better fit to the data obtained with the random simulations (Figure 3D, Figure S2C and S3C), suggesting that the G1/S transition in notochordal cells progresses independently with the cell cycle phase of neighboring cells.

### Model establishment and estimation of the probability of G1/S transition

**Figure 4:**
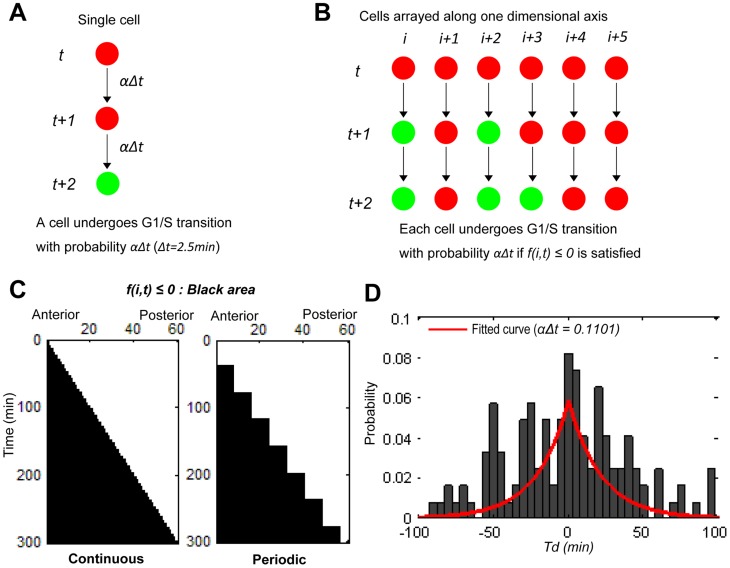
Stochastic modeling of the G1/S transition. (A) Illustration of the stochastic process describing the G1/S transition of a single cell. If the signal comes at time *t*, the cell undergoes its G1/S transition with the probability *αΔt*. Schematically shown is a case in which a given cell does not change its G1 phase at (*t+1*), but rather later, and exhibits its transition to the S phase at (*t+2*) due to the stochastic response of the G1/S transition. (B) Illustration of the stochastic process of cells arrayed along the one-dimensional axis. The signal transmitting function *f(i,t)* was introduced in this case to describe time- and space-dependent cell cycle progression (see Methods). (C) Two dimensional map of *f(i,t)* on the plane of time and space (anterior-posterior axis). The areas satisfying *f(i,t)≤0* and *f(i,t)>0* are filled with black and white, respectively. The continuous and periodic models are defined by setting *z* = 1 and *z* = 8, respectively (see Methods). (D) Searching for the range of the parameter, probability *αΔt*. The probability distribution of the time difference (*Td*) of the G1/S transition between pairs of upper and lower cells was calculated from the experimental data. The red line is a curve fitted according to the least squares method.

In order to unveil a possible regulatory mode of the G1/S transition in notochordal cells, we employed a mathematical modeling approach. The model was constructed according to a Markov process describing the stochastic transition from the G1 phase to the S phase. At each time step *t*, a cell undergoes the G1/S transition with a probability *αΔt* over a short time interval *Δt* (Figure 4A). The spatial position of the notochordal cells is represented as a one-dimensional lattice in the order of anterior to posterior (left to right, respectively, as shown in Figure 4B). Each cell in the lattice is identified by an index *i* (*i* = *1,…,n*). Based on our biological observations described in Figure 1, we assumed the presence of a regulatory wave that conveys a signaling cue to promote the G1/S transition in notochordal cells in a stochastic fashion. This signaling cue on a single dimensional axis is controlled by the signal transmitting function *f(i,t)* of *t* and *i* (Figure 4C). By introducing a tuning parameter *z* in *f(i,t)*, we were able to examine how the probability affects the distinct mode of the regulatory cue (for details, see the Methods section). In other words, *z* corresponds to the step size, which is defined by the width of a given number cells. For example, in the model of *z* = 8, the step size is the total width of eight cells. Based on our observations described above, we assumed modes of continuously traveling waves (continuous mode) and periodically traveling waves (periodic modes) with different periodicity.

In the deterministic cell cycle progression, i.e. *αΔt = 1*, without fluctuation, the continuous wave model (*z* = 1) exhibited a linear mode of progression in the G1/S transition (Figures S4A and S4C). Meanwhile, in the periodic models (a model of *z* = 8 with 30 minutes periodicity is shown in Figure 4C as an example), the G1/S transition progressed in a step-wise mode (Figures S4B and S4C). Under these conditions, the spatial positions of the ARC and PGC always coincided with each other (Figure S4D).

In order to introduce stochasticity into the model, i.e. *αΔt*<1, we estimated the range of probability, *αΔt*. Based on our analysis shown in Figure 3D, the timing of the G1/S transition is not tightly affected by the cell cycle phase of neighboring cells. Therefore, the time differences of S phase entry between the upper and lower cells allowed us to estimate the probability range. A set of time differences in S phase entry was computed for all tracked cells in three individual specimens, and the probability distribution was subsequently obtained by summing all of the data (Figure 4D, histogram). For curve fitting, we theoretically derived the probability distribution function for the time difference based on two independent Bernoulli processes (see the Methods section). By fitting the parameter with experimental data, we estimated that *αΔt* = 0.1101 (Figure 4D, red line), suggesting that the plausible parameter space is located around *αΔt* = 0.1, which specifically means that approximately 10% of cells would exhibit S phase entry in *Δt* = (2.5 minutes) of a single time lap of the imaging transition (see the Methods section). Therefore, we hereafter set the parameter as *αΔt* = 0.1 and examined the system behavior around this value.

### *In silico* simulation revealed that the time interval of PGC reflects the regulatory mode of the stochastic G1/S transition

**Figure 5:**
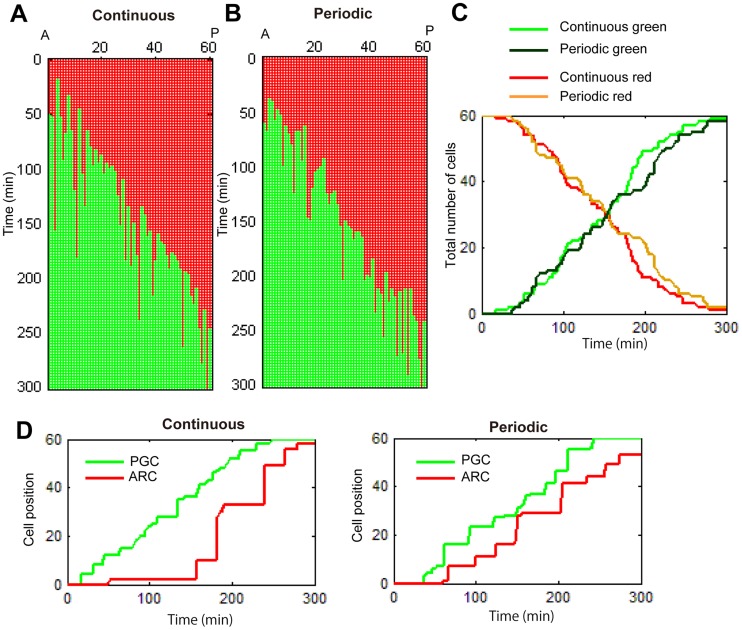
*In silico* simulation reproduces noisy cell cycle progression. (A and B) Two-dimensional map of simulated cell cycle progression on the plane of time and space (anterior-posterior axis). Simulations of the continuous model (*z = 1*) and periodic model (*z = 8*) were implemented. (C) Total number of cells in the G1 and S phases as a function of time, respectively. The green and red lines, and the dark-green and orange lines denote the simulation results obtained by the continuous model and the periodic model, respectively. (D) Positions of the ARC (red line) and PGC (green line) as a function of time, respectively. The results obtained by the continuous and periodic models were drawn individually.

With this parameter setting of G1/S transition probability, the stochastic modes of the G1/S progression were seen in both the continuous and periodic models (Figures 5A and 5B, Movie S3 and S4). Quantification of the temporal changes in the total number of green cells under the continuous (*z* = 1) and periodic (*z* = 8) modes did not show any obvious distinction due to the stochasticity (Figure 5C), as observed in the experimental data analyses shown in Figure 2C.

**Figure 6:**
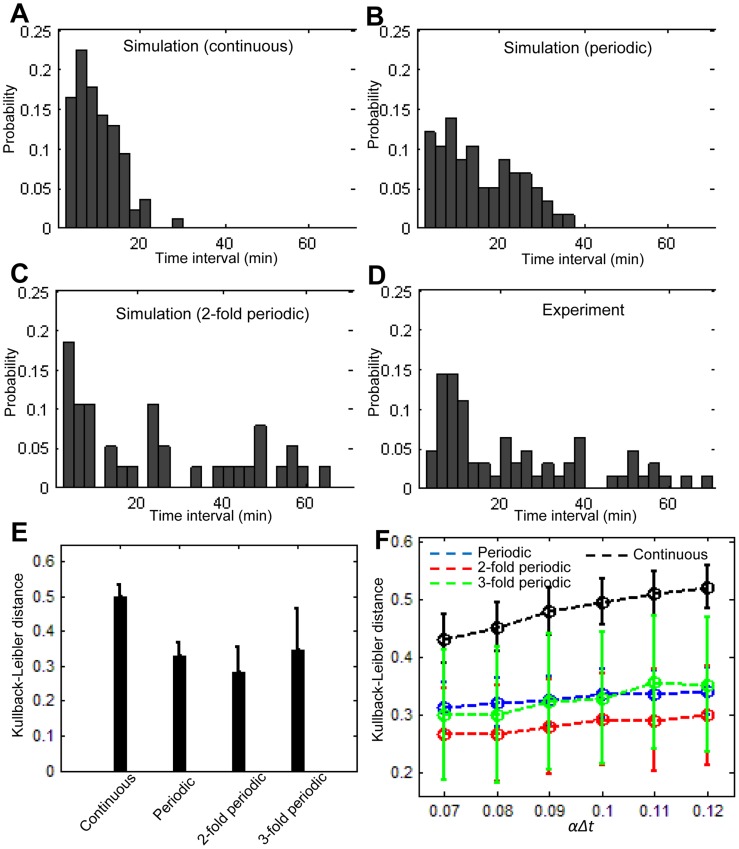
KL distance calculations with a probability distribution of the PGC time interval. (A–C) Probability distribution of the time interval for the PGC (posterior-most green cells) in the continuous model (*z* = 1), periodic model (*z* = 8) and two-fold periodic model (*z* = 16). (D) The distribution of the experiments was calculated by summing all three datasets for the time-lapse imaging. (E) KL distances for four different types of simulation: continuous (*z* = 1), periodic (*z* = 8), two-fold periodic (*z* = 16) and three-fold periodic (*z* = 24). For each model, the KL distance was calculated repeatedly 300 times, and the mean and standard deviation were then calculated. A significance test (Kolmogorov-Smirnov test) was applied for each combination of the KL distance datasets. All combinations exhibited statistical significance, with a p-values of <0.001. (F) The KL distance for six different probability values *αΔt* (*αΔt = 0.07*, *0.08*, *0.09*, *0.1*, *0.11*, *0.12*). For each simulation (circle with error bar), the KL distance was calculated repeatedly 300 times, and the mean and standard deviation were determined.

In order to dissociate the continuous mode from periodic modes regulating the stochastic G1/S transition, we next quantified the positions of the PGC and ARC in these *in silico* simulations. Consequently, the temporal changes in the position of the PGC under the periodic mode appeared to retain its step-wise progression pattern compared to that observed under the continuous mode (Figure 5D, green lines and Figure S5A and S5B), while the position of the ARC exhibited a largely fluctuating pattern under both of these regulatory waves (Figure 5D, red lines, Figure S5C and S5D). Therefore, we decided to perform a detailed analysis of the progressive mode of the PGC position by measuring the time interval from the appearance time of a PGC to that of the next PGC. An example of the calculation procedure of the time interval of the PGC is illustrated in Figure S6. A set of the interval is first computed from a simulation result, after which the probability distribution is obtained. The probability distribution of three different simulations, the continuous model (*z* = 1) (Figure 6A), periodic model (*z* = 8) (Figure 6B) and two-fold periodic model (*z* = 16) (Figure 6C) are subsequently calculated. In the continuous model, the time interval is concentrated on a smaller range (<20 min), and the frequency decreases as the time interval increases. The probability extends to a larger range around 20–40 minutes in the periodic model and extends further and persists up to 60 minutes in the two-fold periodic model. On the other hand, the same analyses of the ARC exhibited no apparent differences between the distributions obtained from three distinct simulation models (Figure S7). Therefore, these analyses indicated that measuring the time interval of PGC reflects the regulatory mode of the stochastic G1/S transition and that the probability distribution function can be used to provide discrimination information in order to analyze the regulatory mode of stochastic changes.

### Model selection predicts the regulatory mode with 60-minute periodicity

We next obtained the probability distribution from the experimental time-lapse imaging data. A set of the time interval was computed from each time-lapse image sequence, and the probability distribution was then calculated by summing all three sets of time-lapse imaging data (Figure 6D). It is obvious that the probability distribution obtained from the experimental data resembles that obtained from the simulated data. In order to quantitatively compare datasets from experiments and simulations, we used the Kullback-Leibler (KL) distance, which measures the difference between two probability distribution functions *p(x)* and *q(x)* (see the Methods section). In order to compare the KL distance between the experiment and simulation, we prepared the probability distribution of the experimental data as *p(x)* and that of the simulated data as *q(x)*, where *x* denotes the time interval of PGC. We computed the KL distance repeatedly, 300 times for four different simulation models, including the continuous (Figure S8A), periodic (Figure S8B), two-fold periodic (Figure S8C) and three-fold periodic (Figure S8D) models, and then calculated the mean and standard deviation of the KL distance (Figure 6E). The mean KL distance values of the periodic models were smaller than those of the continuous model, indicating that the hypothesis of the periodic G1/S transition is likely. Among these periodic models, the two-fold periodic model had the lowest KL distance value. In order to validate our simulations, we carried out sensitivity analyses varying the transition probability *αΔt*, with a range from 0.07 to 0.12 (Figure 6F) and the number of cells constituting a single somite width modified from eight to seven cells (Figure S9); the obtained results did not affect the conclusions. These findings suggest that the G1/S transition progresses under the influence of a periodic regulatory mode and that its periodicity is the most likely to be the two-fold period of somite segmentation.

### Validation of periodicity

**Figure 7:**
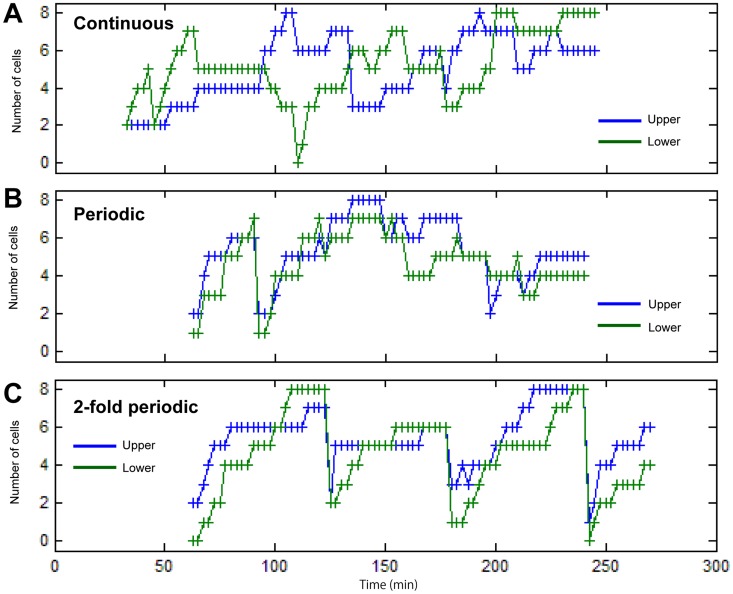
Enumeration of green cells in the G1/S transition window. Total number of green (S) cells in the stochastic window as a function of time for the continuous (A), periodic (B) and two-fold periodic (C) models. The blue and green lines with ‘+’ markers indicate two independent simulation datasets corresponding to the upper and lower sequence data from the experiment, respectively.

In order to provide credible evidence for the periodicity of the regulatory mode of the G1/S transition in notochordal cells, we enumerated the number of green cells in these three distinct periodic simulation models, as we did in the experimental observation shown in Figure 3C. In each simulation model, we performed two independent simulations, which were assumed to involve the upper and lower columns of notochordal cells. According to the continuous model simulation results, the upper and lower columns of cells did not appear to show any obvious synchronized behavior (Figure 7A). In the simulations with the periodic and the two-fold periodic models (Figure 7B and C), two columns of cells exhibited synchronized oscillation. However, in terms of the stability of oscillation, the two-fold periodicity model was more robust than that of the periodic model. Furthermore, the two-fold periodic model appeared to be most suitable for the experimental model shown in Figure 3C.

## Discussion

**Figure 8:**
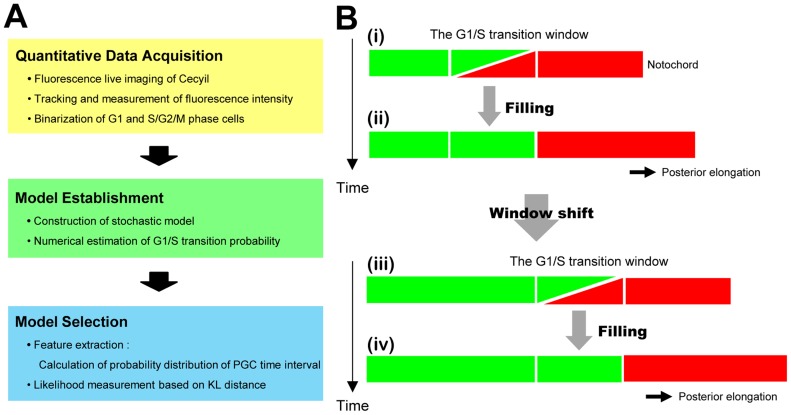
Summary of methodology and findings. (A) Schematic summary of our methodology. Our methodological approach consisted of three successive processes: 1) live imaging and image processing, 2) model establishment and 3) model selection. Each process was further divided into sub-processes, as noted in each box. (B) Schematic illustration of the regulatory mode of the G1/S transition in the developing notochord. (B-i, B-ii) Once the G1/S transition window was established between populations of anterior green (S) and posterior (G1) red cells, the G1 cells in the window stochastically transitioned into the S phase (B-i), after which the window was finally filled with green (S) cells (B-ii). (B-iii, B-iv) The next transition window was established posteriorly adjacent to the previous window. As observed in the previous cycle, an increasing number of green (S) cells filled the window as time progressed (B-iii), until the window was finally filled with green cells (B-iv).

As an inventive application of Fucci technology [48]–[50], the integrative approach we applied in this study was comprised of three parts, including quantitative data acquisition from live imaging, model establishment and model selection (Figure 8A), and revealed that the progressive mode of the G1/S transition window travels in a periodic fashion in newly formed notochordal cells during embryonic axis elongation, as schematically demonstrated in Figure 8b. Once notochordal cells are formed from their precursor pool located at the tip of an embryo, the cells in the G1 phase shown in red are arranged in a line. In the next step, a group of G1 cells enters the S phase. This G1/S transition is temporally stochastic; therefore, a mixed population of green and red cells is established between a group of posterior red cells and a group of anterior green cells, which we described as the G1/S transition window (Figure 8B i). In this window, an increasing number of G1 cells enters the S phase shown in green; therefore, this region is eventually filled with green cells (Figure 8B ii). In the subsequent cycle, the next G1/S transition window shifts a width of 16 cells (two somite widths) posteriorly, thus establishing a new window (Figure 8B iii). The G1 cells in this window enter the S phase in a stochastic manner, finally filling the window (Figure 8B iv).

### Quantitative data acquisition from live imaging

Based on the live imaging data of the developing Cecyil embryos, numerical conversion of the G1/S transition in notochordal cells was carried out using concomitant tracking of the cell fates and changes in the fluorescence intensity of the red and green Fucci signals. As previously described, live imaging of Cecyil embryos provides an almost entire view of morphogenetic cellular movement and cell cycle transition [48]. With careful observation and analyses of morphogenetic cellular events in Cecyil embryos, we noticed that the developing notochord is an appropriate organ for our purposes in establishing a modeling approach. Once notochordal cells are formed, they are arrayed as two columns of cells in the anterior to posterior axis in mid-sagittal optical sections. Their cellular movement is much less active than that of other mesodermal tissues; therefore, the precision of cell tracking and enumeration of spatiotemporal mapping of the G1/S transition are highly ensured.

### Model establishment

In order to establish a reliable mathematical model to extract the regulatory mode of the G1/S transition in developing multicellular tissue, we employed a transition probability model in which cellular states are simply represented as binary states, the G1 or S phase, and temporal variability in S phase entry originates from a random transition with a fixed probability [18]–[22]. The phenomenological model is advantageous, with very few parameters to be measured or inferred in an appropriate manner, although it lacks details in terms of molecular mechanistic insight. The molecular mechanisms underlying the random G1/S transition are currently understood to involve stochasticity in Rb-E2F dynamics, as described by employing stochastic differential equations [37]. This model demonstrates that the stochastic dynamics in the Rb-E2F model can be quantitatively mapped into phenomenological models. Therefore, we decided to apply a simple transition probability model in order to analyze the dynamics of the G1/S transition based on live imaging data of developing multicellular tissue.

We next investigated the independency or dependency of the timing of S phase entry in cells in the G1/S transition window using a linkage analysis. Comparisons of *in silico* simulations and scoring from the imaging data indicated that the G1/S transition in notochordal cells progresses independently from the cell cycle state of neighboring cells. This analysis interestingly suggested that stochasticity in the cellular response is highly intrinsic, even in populations of developing multicellular tissue affected by regulatory surges. This finding firstly confirms the phenomenological concept of the stochastic G1/S transition in *in vivo* tissue development and helped us to estimate the probability of timing of S phase entry, as discussed in the following paragraph.

We then constructed a mathematical model to describe the regulatory mode of the stochastic G1/S transition in notochordal cells. Seeing that the spatial position of the G1/S transition window is always located between newly formed somites and the posterior tip of the embryo, the presence of a sort of progressive developmental wave that triggers S phase entry was postulated and mathematically represented by introducing the signal transmitting function. Our model here has only one free parameter, that is, the probability of timing of the G1/S transition. This parameter was successfully estimated by fitting the theoretically derived probability distribution function with data obtained from live imaging. The notochord structure is profitable in that we simply plotted the time differences in S phase entry between the upper and lower cells in order to estimate the probability range based on the assumption that the developmental wave travels from anterior to posterior and equally to dorsal and ventral cells. Our strategy of model construction with probability estimation is applicable to cell populations in which the timing of the G1/S transition is tightly affected by the cell cycle state of neighboring cells. However, in that case, an alternative method for parameter estimation is required.

### Model selection

With the probability estimation, we conducted model selection by comparing the results obtained from the models with those obtained from the experimental data. We tested several hypothesized models assuming the presence of continuous wave or periodic waves with different periodicity, as reflected in the choice of the tuning parameter *z* of the signal transmitting function. Due to the stochasticity, the boundary of the G1 and S phase cells fluctuated, presenting some difficulty in dissociating the continuous and periodic regulatory mode. For instance, merely counting the temporal changes in the total number of green and red cells did not provide any information about the spatial progressive mode of the G1/S transition window. It is probable that the noise strength numerically estimated in this work completely veiled the spatial information related to a possible regulatory mode in this analysis. Therefore, we focused on the PGC (posterior-most green cell) and ARC (anterior-most red cell). After conducting several trials, the probability distribution in the time interval of the PGC, but not the ARC, was found to provide information regarding a possible regulatory mode. Once the G1/S transition window opens at a given time point of embryonic development, all cells within the window equally acquire the chance to enter the S phase. Among these cells, the cells that immediately traverse the G1/S transition demarcate the spatial position of the newly established window. Therefore, the PGC tends to indicate the posterior end of the window, although this is not always precise, thus implying some information about the possibility of retrieving the progressive mode of the window shift. In contrast to the PGC, the ARC comprises the last cells to traverse the G1/S transition affected by the window at a given time, thus losing the spatio-temporal information of the window. This finding highly contributed to our model selection, which suggests that analyses of immediately responding cells provide clues for inferring possible regulatory modes under stochastic cellular behavior. In order to statistically determine which model represents the most likely scenario, we applied the KL distance, a measurement of the difference between two probability distributions [51]–[53]. The KL distance is applied as a discriminant function; therefore, it is often used as a basic tool for model inference and selection. This use of the KL distance enables hypothesized models to be ranked from likely to unlikely, thus allowing for the selection of the most possible regulatory mode. Consequently, the G1/S transition in notochordal cells was found to progress under the influence of periodic developmental waves, and its periodicity is most likely to involve the two-fold period of somite segmentation. We validated this result by employing another method to score the results of the *in silico* simulation. Enumeration of the total number of green cells in the G1/S transition window was applied, as performed in the linkage analysis to analyze the independency of the timing of S phase entry in each cell. The analysis also demonstrated that only periodic waves of the two-fold period of somite segmentation showed stable synchronized oscillation.

### Stepwise posterior shift of the G1/S transition window

Our analyses also clarified that the G1/S transition in notochordal cells posteriorly travels in a stepwise fashion, which appears to be accompanied by embryonic body axis elongation and neighboring paraxial mesoderm segmentation. This finding implies that the G1/S transition regulatory window is reminiscent of the bistability window of the mutual inhibition of FGF and RA signaling, which has been proposed to provide a poised state of segmentation of the paraxial mesoderm as well as initial differentiation of neuronal cells from stem cells [54]–[58]. Within the bistability window, cells can be triggered to switch between either of the two steady states. This trigger is provided by the periodic signal of the segmentation clock in the paraxial mesoderm. In fact, the occurrence of periodic surges of the G1/S transition of paraxial mesoderm cells at the exact position of the bistability window in chick development has been proposed [7], although we did not observe a similar phenomenon in the zebrafish paraxial mesoderm [48]. However, the periodicity operated in the G1/S transition window is two-fold longer than that of the zebrafish segmentation clock. None of the cyclic patterns of the gene expression have been observed in early notochordal cell development. It has also not yet been elucidated how such an extrinsic bistable switch comprised by FGF and RA gradients affects the intracellular machinery. Therefore, the molecular machinery of the cyclic regulation of the G1/S transition in notochordal cells remains to be elucidated.

As it has been reported that a component of the segmentation clock oscillates during chick fore-limb development with a six-hour periodicity that is four-fold longer than that of chick somite segmentation [59], a mechanism that multiplies the periodicity composed by Notch signals may exist in developing tissues, potentially linked to periodic surges of cell division, as previously described [3]. Given that intracellular bistable switches have also been proposed to be involved in the cell cycle phase transitions [30], [36], it is interesting to consider how distinct levels of multiple bistable switches interact to induce dynamic changes in the cellular properties, which may cause phenomenologically described complex modes of periodic entrainment. It would be worthwhile to analyze a model organism in which the activity of intracellular molecules composing a bistable switch, such as Rb and E2F, are genetically modified.

Recently, a Doppler effect has been reported to be involved in zebrafish segmentation [60]. Time-lapse observations of the transcriptional activity of *her1*, a component of the segmentation clock, during embryonic development revealed that its oscillation in the posterior portion is slower than that in the anterior part. Since the anterior oscillation is directly linked to the pace of segmentation, the shortening rate of the presomitic mesoderm due to a gradual slowdown in the rate of embryonic body axis elongation may affect the segmentation pace through a Doppler effect. It is possible that the stepwise posterior shift of the G1/S transition window observed in this work was also affected by this Doppler effect. However, this is less obvious, because the G1/S transition window is always located between newly formed segments and the tail tip throughout embryonic development.

### Stochasticity in the G1/S transition and its significance

It is relevant to consider how the stochastic G1/S transition would benefit organ development. Vertebrate embryos employ a robust system for developmental pattern formation, with the primary function of establishing a reproducible pattern in a proliferating population of cells. Biological robustness is proposed to be a characteristic required to maintain cell functions in the face of external and internal perturbations [61]. The stochasticity observed in molecular and cellular functions can be considered an opposite concept to robustness. For instance, cell proliferation has been reported to serve as a source of noise for the synchronized expression of genes of the segmentation clock [16]. However, stochasticity in the cell cycle also provides the mechanical flexibility necessary to adapt to a fluctuating environment and/or respond to sudden changes in environmental cues. Therefore, it is possible that the stochasticity of the G1/S transition in notochordal cells is required to maintain the tissue structure and provide a poised state for subsequent tissue development.

### Implications in pathophysiological tissue development based on quantitative imaging and mathematical modeling

Correlations of cell cycle progression in embryonic morphogenesis and organ development have long been proposed based on counts of the number of cells exhibiting mitotic nuclei in tissue sections or three-dimensionally reconstructed fixed tissues. By exploiting fluorescent live imaging of Cecyil embryos with subsequent scoring and mathematical modeling, we successfully extrapolated an unprecedented regulatory pattern of the G1/S transition during early notochord development. It is possible that periodic regulation of cell cycle progression in a group of cells is a common phenomenon and that the stochastic nature of cell cycle progression in developing tissue hinders an unobserved regulatory system. Our approach provides a way to infer the regulatory mode of the stochastic G1/S transition in tissue and organ development. The potential utility of this approach may be applicable, not only for understanding physiological development, but also clarifying mechanisms of pathological tissue development, such as that involving carcinogenesis. Generally, cancer cells are associated with the affected cell cycle mechanism, which may directly or indirectly influence the bistable switch of cell cycle transitions. Therefore, tumor tissue growth can increase the occurrence of the complex or de-regulated mode. The gold standard for cancer classification fundamentally involves microarray-based gene profiling to characterize the cancer signature. The utility of mathematical models of stochastic molecular pathways is also proposed to be applicable for cancer diagnosis [37]. Mathematical models based on experimental studies are proposed to bridge genetics and tumor behavior, with the potential to provide better personalized cancer treatment [62], [63]. The development of live imaging-based mathematical models to infer both the heterogeneity and dynamics of cellular behavior will provide further insight into the characteristic features of both tumor and physiological tissue development.

## Materials and Methods

### Ethics statement

All experimental procedures using zebrafishes were conducted with following ethical regulations of the Experimental Animal Committee of RIKEN BSI.

### Live imaging of Cecyil embryos

*In vivo* time-lapse imaging was carried out as previously described [48], with some modifications. Dechorionated embryos were embedded in small holes molded by glass beads (Iuchi BZ-1) on the surface of 1% agarose (Takara L03) solution in E3 medium (5 mM NaCl, 0.17 mM KCl, 0.4 mM CaCl2 and 0.16 mM MgSO4) and then covered with 0.3% agarose. The chamber encasing the embedded embryos was filled with E3 solution containing Tricaine. Time-lapse 3D imaging was subsequently performed in the xyz-t mode using a confocal upright microscope EZ-C1 system (Nikon, Tokyo) equipped with a water-immersion 16× objective (N.A. 0.8). Two laser lines, 488 nm and 561 nm, were used. Differential interference contrast microscope (DIC) images were concomitantly acquired. The recording interval was 2.5 minutes. At each time point, optical sagittal sections of 27∼34 confocal images along the *z* axis were acquired to access developing notochord tissue located in the deepest embryonic layer. In order to avoid cross-detection of green and orange signals, the images were acquired sequentially at 488 nm and 561 nm. Total eighteen time-lapse imaging data of distinct embryos were obtained. Seven of them were subjected to image processing, and representative data of three distinct embryos were used for modeling analyses.

### Confocal observation of the fixed embryos

Dechorionated Cecyil embryos were fixed with 4% PFA (pH 7.4) in PBS for one hour at room temperature, then washed in 0.1% Triton in PBS (PBT). The embryos were then incubated one hour at room temperature in PBT containing 660 nM of Alexa Fluor 647-Phalloidin and washed in 0.1% Triton in PBS (PBT). Image acquisition was performed using an EZ-C1 (Nikon) confocal upright microscope system equipped with 488 nm, 561 nm and 638 nm laser lines. More than twenty distinct fixed embryos were observed with comparing to time-laps data.

### Image processing

Optical slices focusing on tissue layers containing notochord tissue at each time point were manually selected, and the time sequence was reconstructed for further image processing. Cell tracking was performed using the Manual Tracking plugin in the ImageJ software program. To ensure recognition of the final position of each notochordal cell, the tracking was conducted in a temporally backward and forward manner. In order to monitor temporal changes in the green and red fluorescence intensity of each cell, we developed a program that automatically averages the signal intensity of a defined area of the nucleus at each time point on sequential images. This program was written in C/C++ for use under Win32 environments. In order to define the timing of the G1/S transition of each cell, the time course of the signal intensity of the green and red fluorescence signals was plotted. The time point of transition was defined as the time at which the intensity of the green signal increased and exceeded that of the red signal continuously five times points.

### Linkage analysis of S phase entry

An algorithmic description for the random and biased simulations of the binary valued cell distribution was provided. For the random case, we spatially distributed the upper and lower green cells randomly, while keeping the same number of green cells as observed in the experiments. The initial state was set to all red. With respect to the randomly chosen site *i*, only if the chosen site was red, the site was set to green, and this procedure was repeated until the total number of green cells reached the experimentally observed number. The expectation value of the number of green cell pairs was calculated by repeating the simulation 10,000 times. For the biased case, we first distributed the upper green cells randomly as performed in the random case. We then distributed the lower green cells according to which (randomly chosen) site was set to green, with a probability *q* (here *q* = 0.9 is taken) when the corresponding upper cell was green and a probability *1-q* ( = 0.1) when the corresponding upper cell was red. The expectation value of the number of green cell pairs was calculated by repeating the simulation 10,000 times.

### Mathematical modeling of the stochastic G1/S transition

In order to describe the stochastic cell cycle transition, we developed a mathematical model based on the Markov process [64], [65]. When only a single cell is considered, at each time step *t*, a cell undergoes the G1/S transition with a probability *αΔt* within a short time interval *Δt* (Figure 4A). The backward process of the transition, such as the green (S) to red (G1) transition, is not assumed. The time *t* is measured in units corresponding to the time-lapse interval *Δt* = 2.5 minutes, and the parameter *α* represents the probability of the transition per unit time *Δt*. In a developing notochord, the cells are arrayed along the anterior-posterior axis, and the cell cycle transition progresses from anterior to posterior in a time-dependent manner. In order to model this time-dependent stochastic process on the one-dimensional axis, we introduced the signal transmitting function *f(i,t)* of *t* and *i*, where *i* denotes the cell position along the anterior-posterior axis. The stochastic process is designed such that each cell independently undergoes the G1/S transition with a probability *αΔt*, if the condition *f(i,t)≤0* is satisfied (Figure 4B). The signal transmitting function *f(i,t)* can be written in the following form:
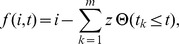
where *z* is a positive integer, and *t_k_* is taken as *t_k_ = k L_c_ z/(λΔt). L_c_* is the diameter of notochordal cells and *λ* is the posterior elongation speed, both of which are estimated from time-lapse imaging data as *L_c_* = 5 µm and *λ* = 4/3 µm/min, respectively. Therefore, the number of cells constituting a single somite width is calculated as T*λ/L_c_ = 8, where *T* ( = 30 min) is a period of the segmentation clock. The time-dependent function 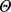 *(t_k_≤t)* is a step function satisfying
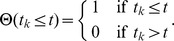
In the numerical simulations, *t_m_* should be greater than the maximum simulation time *tmax*; therefore, *m* should be taken so as to be *m>m_c_ = λΔt tmax/(L_c_z)*.

Hypothesized models can be obtained by modulating the parameter *z*. We categorized the models into two basic types, “the continuous model” and “the periodic model.” If we take *z* = 1, we can obtain the continuous model in which the external cell cycle cue comes continuously in a spatiotemporally correlated manner. The periodic models are further divided into subtypes based on the cue period. In this case, *z* can be interpreted as the number of cells constituting a single somite width. Hence, if we take *z* = 8, we can obtain the (normal) periodic model in which the signaling cue comes periodically, in concert with the cycle of the segmentation clock. When *z* is taken to be equal to the integral multiple of 8, we have a multifold periodic model in which the period is a multiple of the cycle of the segmentation clock. In this study, we considered the two-fold periodic model (*z* = 16) and three-fold periodic model (*z* = 24). Regions satisfying *f(i,t)≤0* for both the continuous and periodic models are depicted in Figure 4C.

### Probability distribution function for differences in the S phase entry time

We described how the probability distribution function for the difference in the S phase entry time between two independent cells was derived. We considered a Bernoulli trial with a probability *p* of success. A variable *x* is represented as *x* = (0,1), thus *x* takes 1 (success) with probability *p* and *x* takes 0 (failure) with probability *1-p*. The initial value of *x* is set to 0, and the trial is repeated until we have the first success *x* = 1. The probability distribution function *f(t)* that *x* becomes 1 at the trial time *t* is given by
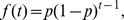
where *f(t)* satisfies
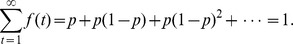



We next considered the joint probability distribution *f(t_1_,t_2_)* of two independent trials,
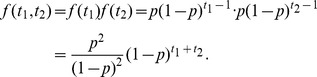
The two independent processes correspond to experimentally obtained processes of the G1/S transition for an upper and lower cell. In order to derive the distribution function for time difference *t = t_1_−t_2_*, we summed *f(t_1_,t_2_)* fixing *t* with respect to *t_1_* and *t_2_*,
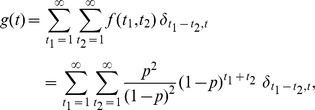
where *δ_t, t′_* is the Kronecker delta. With a short calculation, we obtained the analytical expression of *g(t)*

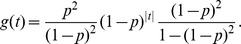
This expression was used to estimate the parameter *p = αΔt*, the probability of the G1/S transition per unit time *Δt* (Figure 4D).

### Kullback-Leibler distance

We utilized the Kullback-Leibler (KL) distance to measure the difference between the experimental and simulation data and determine which hypothesis best reflects the system. The KL distance [51] is defined as
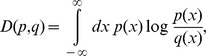
where *p(x)* and *q(x)* denote probability distribution functions. In our calculations, *p(x)* represents the probability distribution function of time interval for PGC obtained from the experimental data and *q(x)* represents that obtained from the simulation data. By definition, if *p(x) = q(x)*, obviously *D(p,q) = 0*.

### Numerical simulations

The numerical simulations and statistical analyses were carried out using the MATLAB software program (The Mathworks, Inc.). Parameter fitting was performed with the MATLAB software using the fminsearch function. The target function was chosen as the residual sum of squares between theoretically estimated values and the experimentally obtained values.

## Supporting Information

Figure S1 **Definition of the stochastic G1/S transition window.** The stochastic G1/S transition window is defined as eight cells composed of seven cells anterior to the PGC (posterior most green cell), as well as the PGC. In this schematic drawing, the lower PGC is taken as a landmark to define the window indicated by the blue box.
(TIF)

Figure S2 **Systematic analyses of binarized images of G1/S cell cycle progression for sample #2.** The same analyses demonstrated in Figure 3 for sample #2 are shown. (A) Positions of the ARC and PGC as a function of time. The upper and lower sequences of notochordal cells along the anterior-posterior axis are drawn individually. (B) Total number of green cells in the G1/S transition window as a function of time. The blue and green lines with ‘+’ markers indicate the upper and lower sequence data, respectively. (C) Number of green cell pairs in the G1/S transition window as a function of time. The red ‘+’ markers indicate the data obtained from the experimental results. The black and blue lines indicate data obtained using random and biased simulation, respectively.
(TIF)

Figure S3 **Systematic analyses of binarized images of G1/S cell cycle progression for sample #3.** The same analyses demonstrated in Figure 3 for sample #3 are shown. (A) Positions of the ARC and PGC as a function of time. The upper and lower sequences of notochordal cells along the anterior-posterior axis are drawn individually. (B) Total number of green cells in the G1/S transition window as a function of time. The blue and green lines with ‘+’ markers indicate the upper and lower sequence data, respectively. (C) Number of green cell pairs in the G1/S transition window as a function of time. The red ‘+’ markers indicate the data obtained from the experimental results. The black and blue lines indicate data obtained using random and biased simulation, respectively.
(TIF)

Figure S4 **Spatiotemporal pattern of deterministic cell cycle progression.** (A and B) Two-dimensional map of simulated cell cycle progression on the plane of time and space (anterior-posterior axis). Simulations of the continuous model (*z = 1*) and periodic model (*z = 8*) were implemented. (C) The total number of cells in the G1 (red) and S (green) phases as a function of time. The solid and broken lines denote the simulation results of the continuous model and the periodic model, respectively. (D) Positions of the ARC and PGC as a function of time. The solid and broken lines denote the simulation results of the continuous and periodic models, respectively. The ARC line overlaps the PGC line.
(TIF)

Figure S5 **Overwriting of the ARC and PGC for six repeated simulations.** (A and C) Positions of the ARC (A) and PGC (C) as a function of time in the continuous model. (B and D) Positions of the ARC (B) and PGC (D) as a function of time in the periodic model. The progressive pattern of the PGC well represents the regulatory mode.
(TIF)

Figure S6 **Example of time interval calculation for the PGC.** The cell located at *i+2* enters its S phase at *T1 = t+1*, indicating that this cell is recognized as the PGC at this time point. The cell located at *i+3* enters its S phase at *T2 = t+3*, indicating that this cell is now recognized to be the next PGC. In this example, the time interval for the PGC is calculated as the difference of *T1* and *T2*, i.e. the waiting time = *T2−T1* = *2*.
(TIF)

Figure S7 **The results of an analysis of the time interval from the appearance of the ARC (anterior-most red cells) to that of the next ARC.** (A–C) The probability distribution of the time intervals for the ARC in the continuous model (*z* = 1), periodic model (*z* = 8) and two-fold periodic model (*z* = 16).
(TIF)

Figure S8 **Probability distribution of the KL distance.** Histograms of the computed KL distance for four different simulation models: the continuous (A), periodic (B), two-fold periodic (C) and three-fold periodic (D) models. The KL distance was calculated 300 times for each simulation.
(TIF)

Figure S9 **KL distances obtained under the assumption that the somite length corresponds to seven notochordal cells.** The KL distances for the four types of simulation: the continuous (*z* = 1), periodic (*z* = 7), two-fold periodic (*z* = 14) and three-fold periodic (*z* = 21) models. For each model, the KL distance was calculated 300 times, and the mean and standard deviation were determined.
(TIF)

Movie S1 **Live imaging of the developing notochord in a Cecyil embryo.** Optical slices of a mid-sagittal section of the posterior portion are shown. The G1/S transition of notochordal cells is demonstrated by a fluorescence color switch of red to green. The G1/S transition window travels posteriorly along the body axis. Left to anterior, right to posterior. Dorsal to up, ventral to bottom.
(AVI)

Movie S2 **Time-lapse movie for binarized images of notochordal cells obtained from an experiment.** The binary valued cellular states of the upper (dorsal) and lower (ventral) columns of cells are plotted on the anterior (left) to posterior (right) axis. Cells in the G1 and S phases are indicated by red and green points, respectively.
(AVI)

Movie S3 **Time-lapse movie for** ***in silico*** **simulation with continuous waves.** The binary valued cellular states of the upper and lower columns of cells are plotted on the anterior (left) to posterior (right) axis. In order to display the upper and lower columns of cells, two independent simulations were performed. Cells in the G1 and S phases are indicated by red and green points, respectively.
(AVI)

Movie S4 **Time-lapse movie for** ***in silico*** **simulation with periodic waves.** The binary valued cellular states of the upper and lower columns of cells are plotted on the anterior (left) to posterior (right) axis. In order to display the upper and lower columns of cells, two independent simulations were performed. Cells in the G1 and S phases are indicated by red and green points, respectively.
(AVI)
