# Supplementary figures and images for: Live Imaging-Based Model Selection Reveals Periodic Regulation of the Stochastic G1/S Phase Transition in Vertebrate Axial Development
Source: PLoS Comput Biol. 2014 Dec 4;10(12):e1003957. doi: 10.1371/journal.pcbi.1003957 (PMC4256085; doi:10.1371/journal.pcbi.1003957)

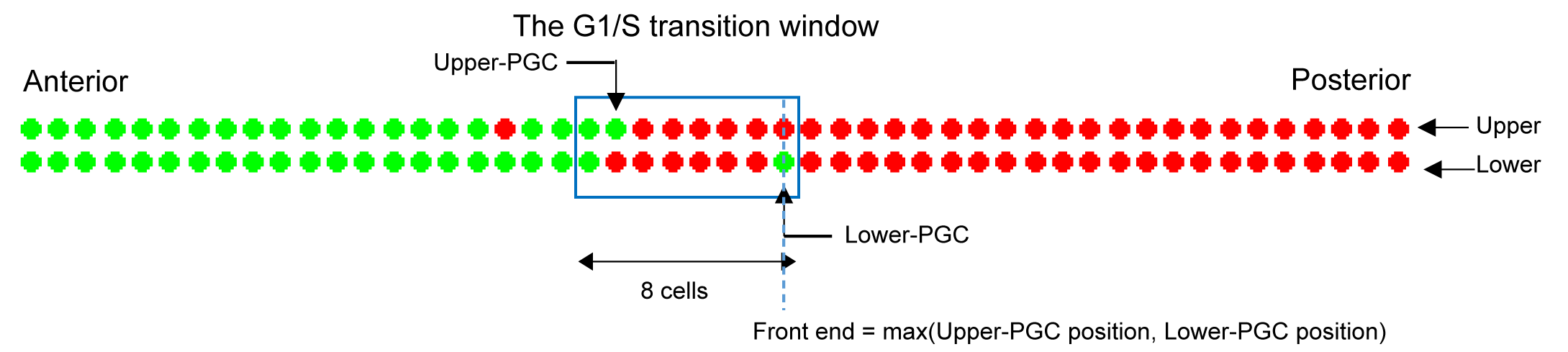

Supplement: Figure S1 — Definition of the stochastic G1/S transition window. The stochastic G1/S transition window is defined as eight cells composed of seven cells anterior to the PGC (posterior most green cell), as well as the PGC. In this schematic drawing, the lower PGC is taken as a landmark to define the window indicated by the blue box. (TIF) [file pcbi.1003957.s001.tif]

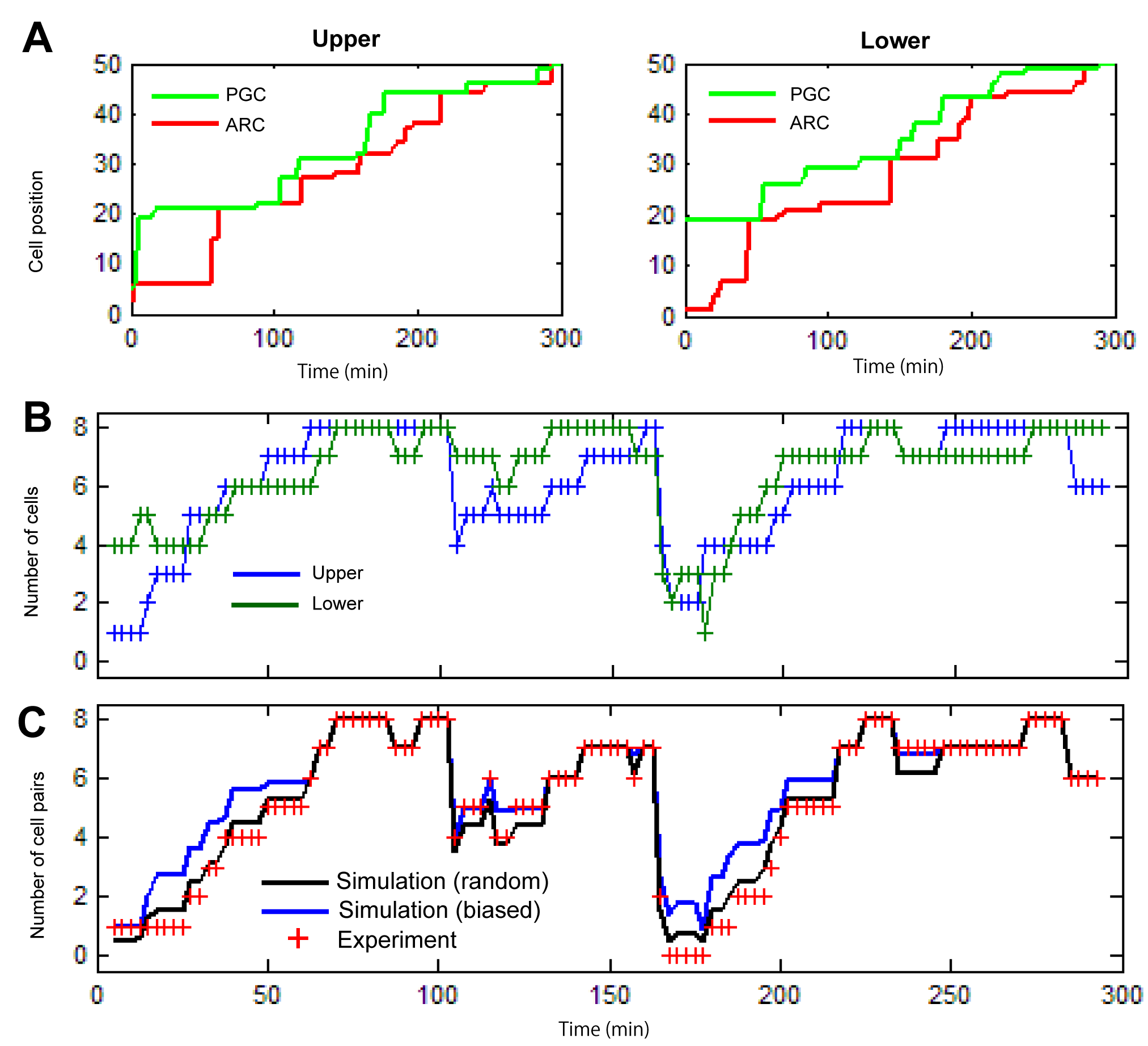

Supplement: Figure S2 — Systematic analyses of binarized images of G1/S cell cycle progression for sample #2. The same analyses demonstrated in Figure 3 for sample #2 are shown. (A) Positions of the ARC and PGC as a function of time. The upper and lower sequences of notochordal cells along the anterior-posterior axis are drawn individually. (B) Total number of green cells in the G1/S transition window as a function of time. The blue and green lines with ‘+’ markers indicate the upper and lower sequence data, respectively. (C) Number of green cell pairs in the G1/S transition window as a function of time. The red ‘+’ markers indicate the data obtained from the experimental results. The black and blue lines indicate data obtained using random and biased simulation, respectively. (TIF) [file pcbi.1003957.s002.tif]

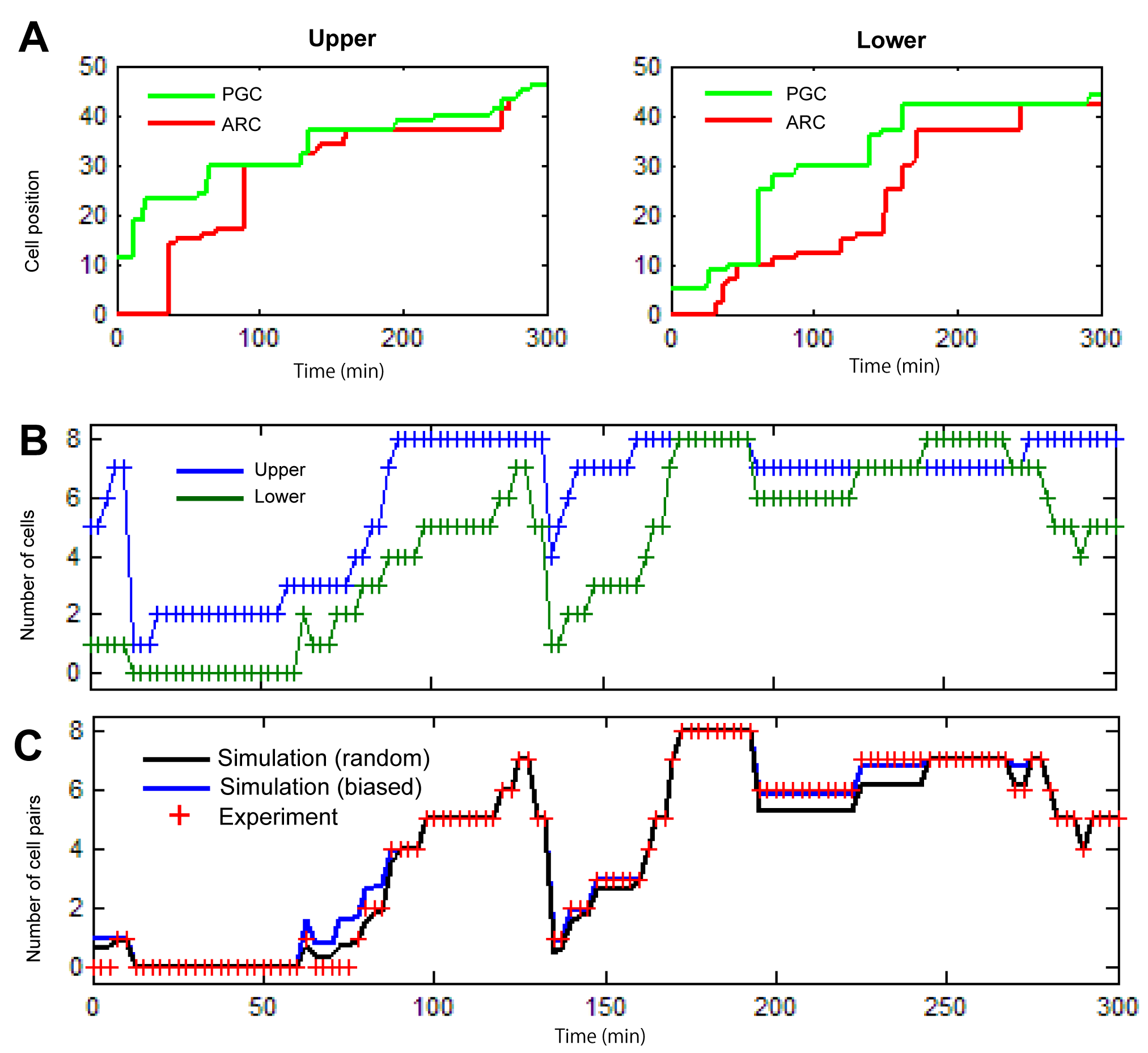

Supplement: Figure S3 — Systematic analyses of binarized images of G1/S cell cycle progression for sample #3. The same analyses demonstrated in Figure 3 for sample #3 are shown. (A) Positions of the ARC and PGC as a function of time. The upper and lower sequences of notochordal cells along the anterior-posterior axis are drawn individually. (B) Total number of green cells in the G1/S transition window as a function of time. The blue and green lines with ‘+’ markers indicate the upper and lower sequence data, respectively. (C) Number of green cell pairs in the G1/S transition window as a function of time. The red ‘+’ markers indicate the data obtained from the experimental results. The black and blue lines indicate data obtained using random and biased simulation, respectively. (TIF) [file pcbi.1003957.s003.tif]

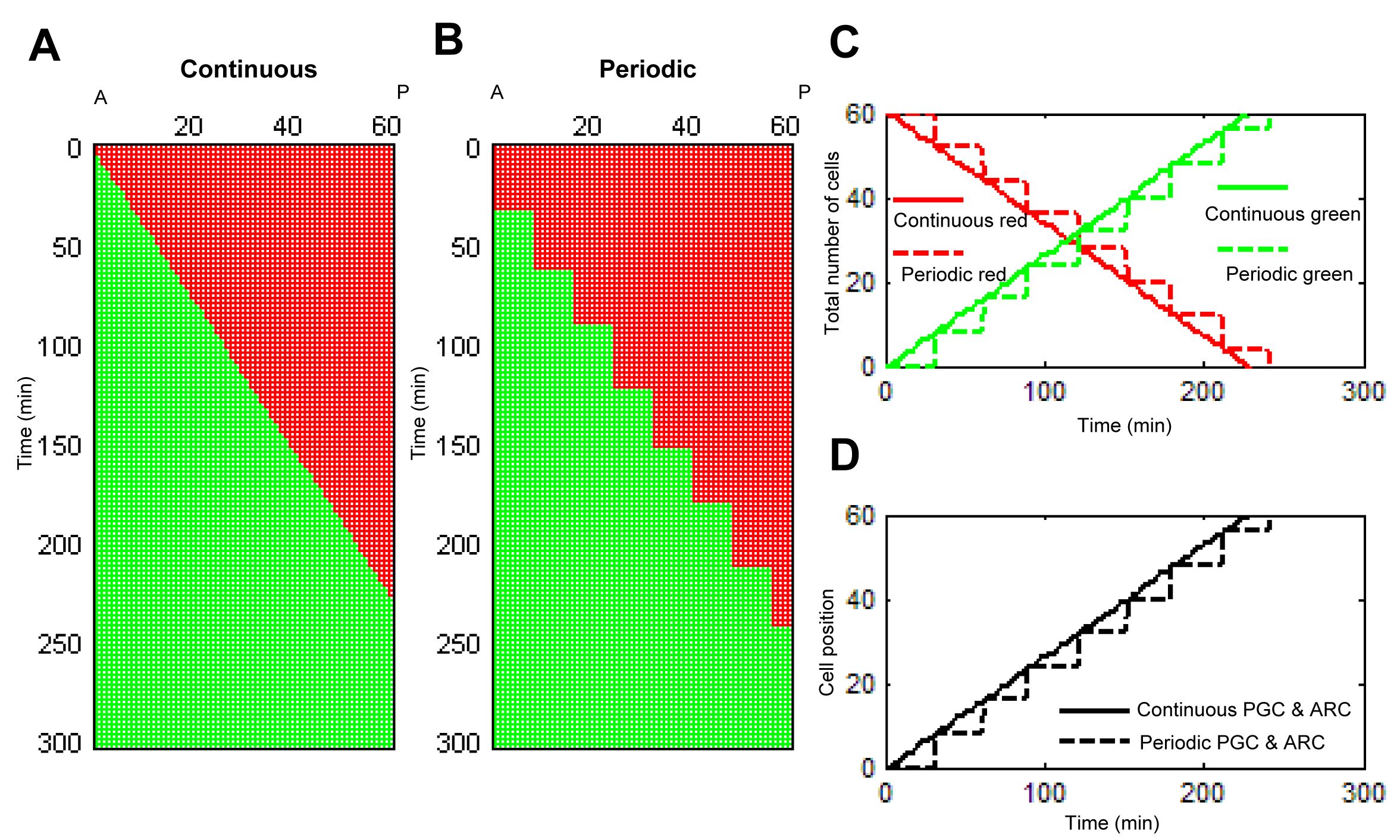

Supplement: Figure S4 — Spatiotemporal pattern of deterministic cell cycle progression. (A and B) Two-dimensional map of simulated cell cycle progression on the plane of time and space (anterior-posterior axis). Simulations of the continuous model (z = 1) and periodic model (z = 8) were implemented. (C) The total number of cells in the G1 (red) and S (green) phases as a function of time. The solid and broken lines denote the simulation results of the continuous model and the periodic model, respectively. (D) Positions of the ARC and PGC as a function of time. The solid and broken lines denote the simulation results of the continuous and periodic models, respectively. The ARC line overlaps the PGC line. (TIF) [file pcbi.1003957.s004.tif]

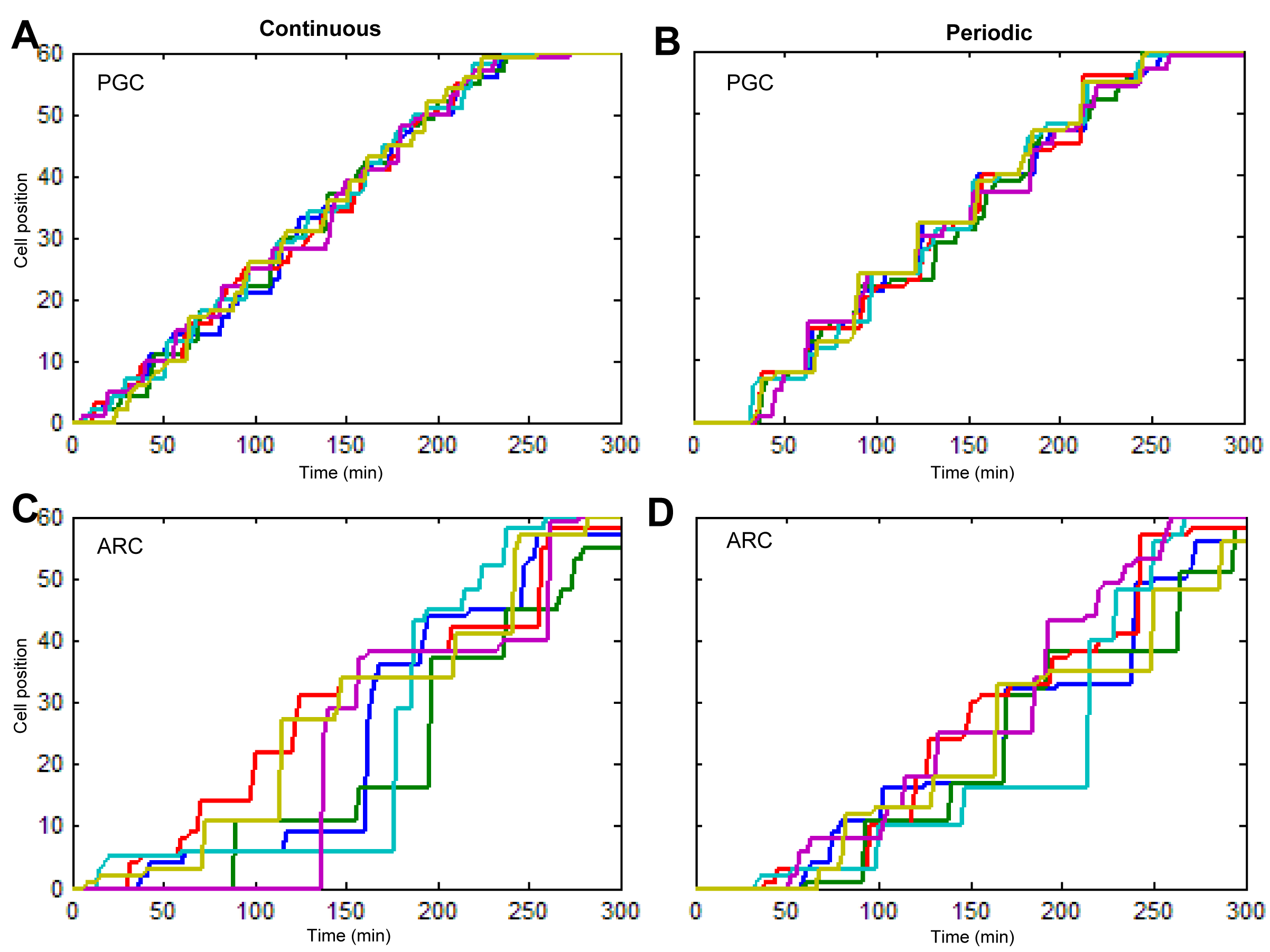

Supplement: Figure S5 — Overwriting of the ARC and PGC for six repeated simulations. (A and C) Positions of the ARC (A) and PGC (C) as a function of time in the continuous model. (B and D) Positions of the ARC (B) and PGC (D) as a function of time in the periodic model. The progressive pattern of the PGC well represents the regulatory mode. (TIF) [file pcbi.1003957.s005.tif]

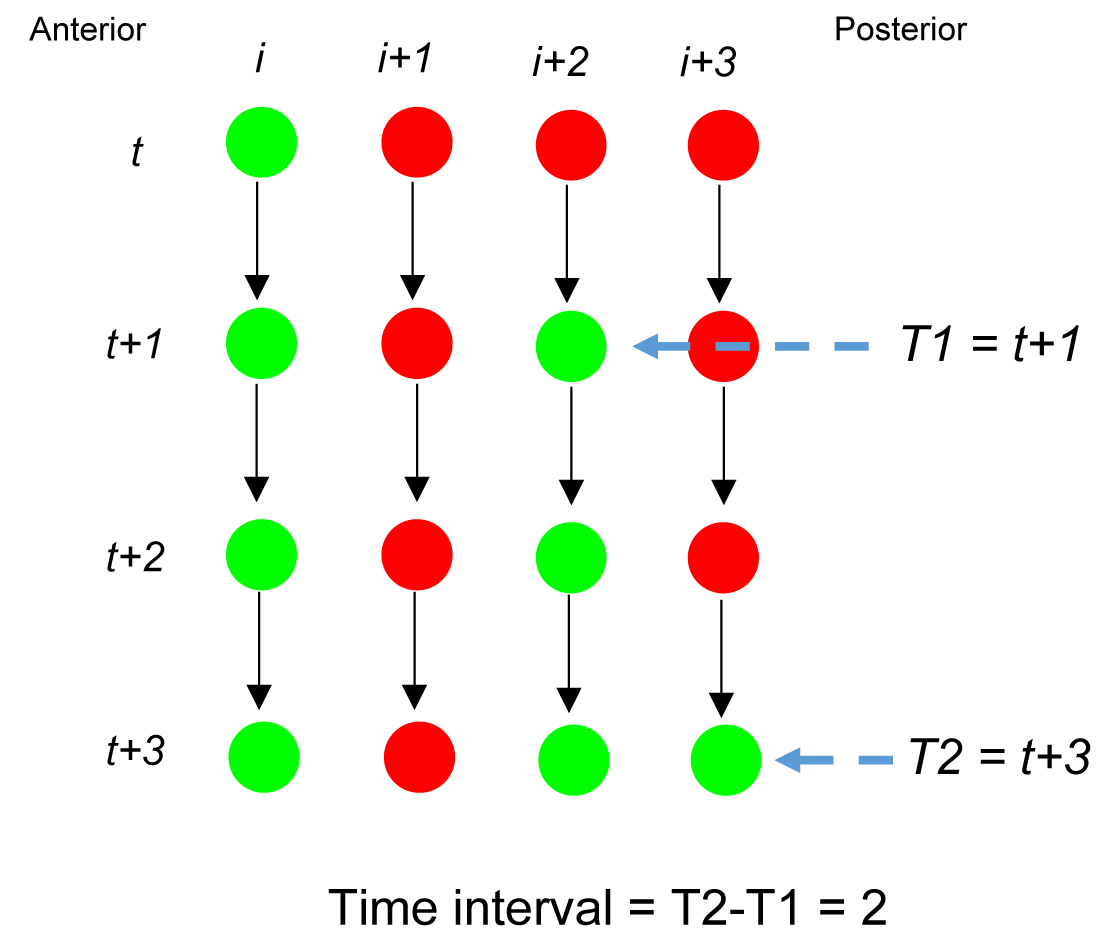

Supplement: Figure S6 — Example of time interval calculation for the PGC. The cell located at i+2 enters its S phase at T1 = t+1, indicating that this cell is recognized as the PGC at this time point. The cell located at i+3 enters its S phase at T2 = t+3, indicating that this cell is now recognized to be the next PGC. In this example, the time interval for the PGC is calculated as the difference of T1 and T2, i.e. the waiting time = T2−T1 = 2. (TIF) [file pcbi.1003957.s006.tif]

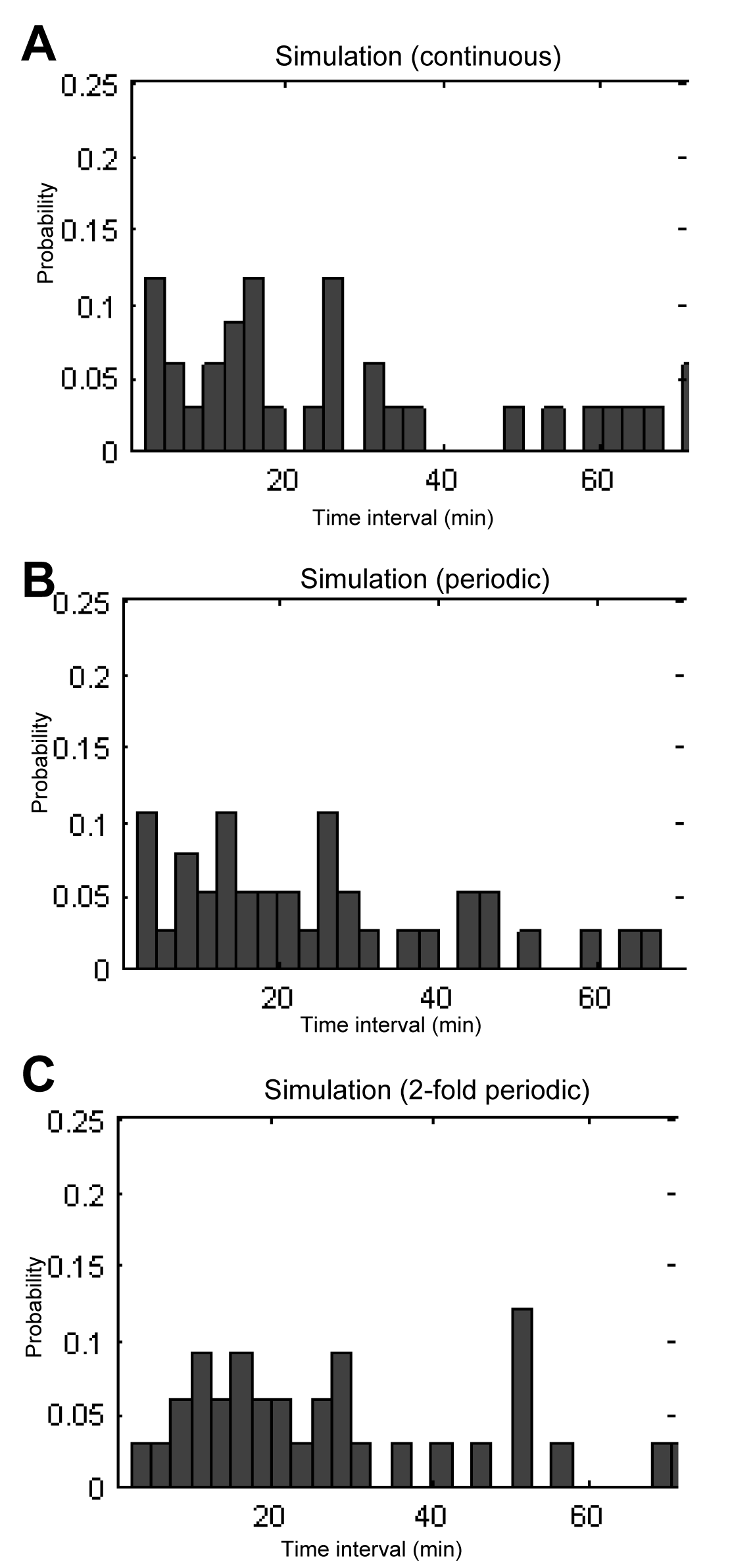

Supplement: Figure S7 — The results of an analysis of the time interval from the appearance of the ARC (anterior-most red cells) to that of the next ARC. (A–C) The probability distribution of the time intervals for the ARC in the continuous model (z = 1), periodic model (z = 8) and two-fold periodic model (z = 16). (TIF) [file pcbi.1003957.s007.tif]

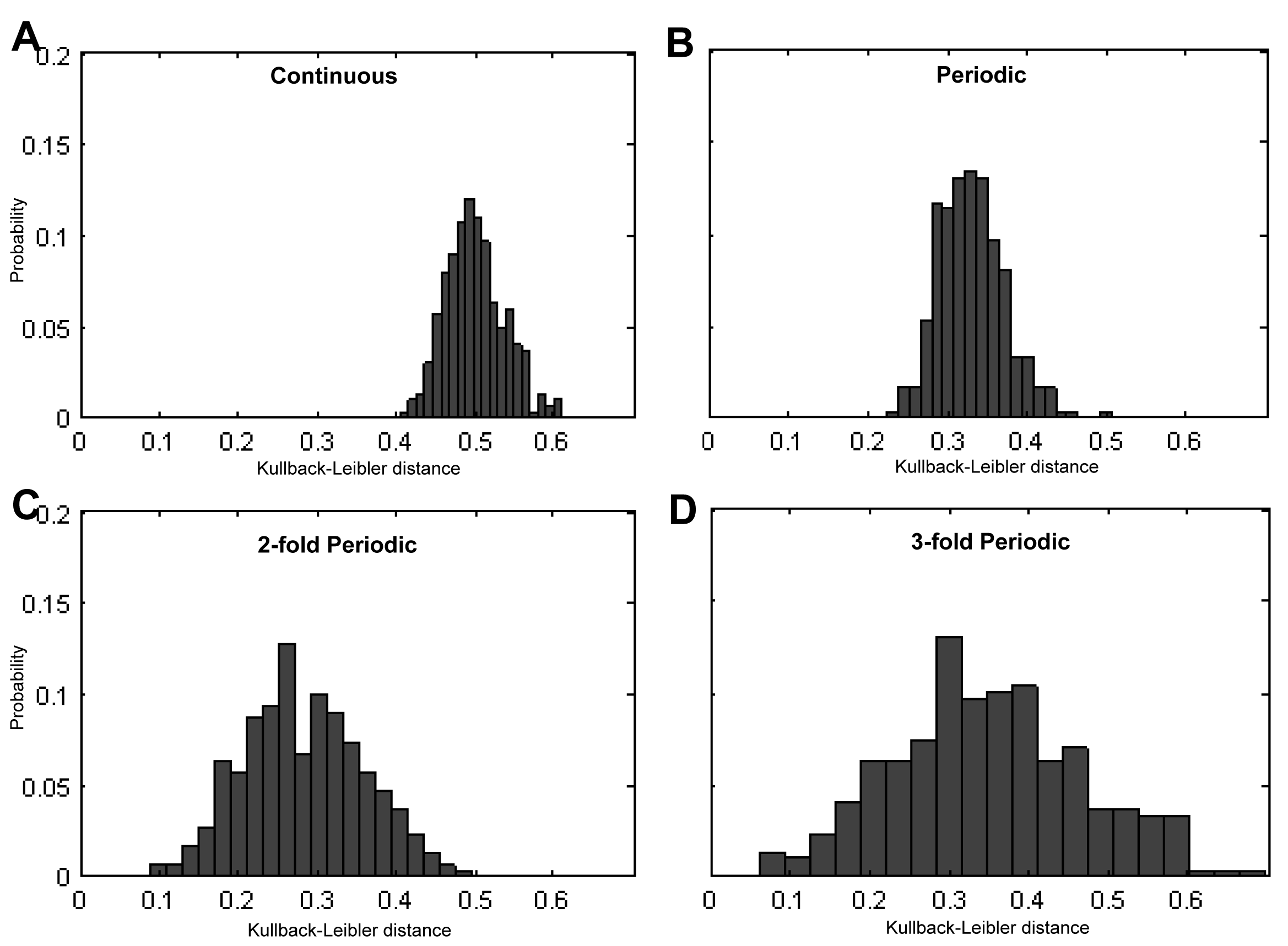

Supplement: Figure S8 — Probability distribution of the KL distance. Histograms of the computed KL distance for four different simulation models: the continuous (A), periodic (B), two-fold periodic (C) and three-fold periodic (D) models. The KL distance was calculated 300 times for each simulation. (TIF) [file pcbi.1003957.s008.tif]

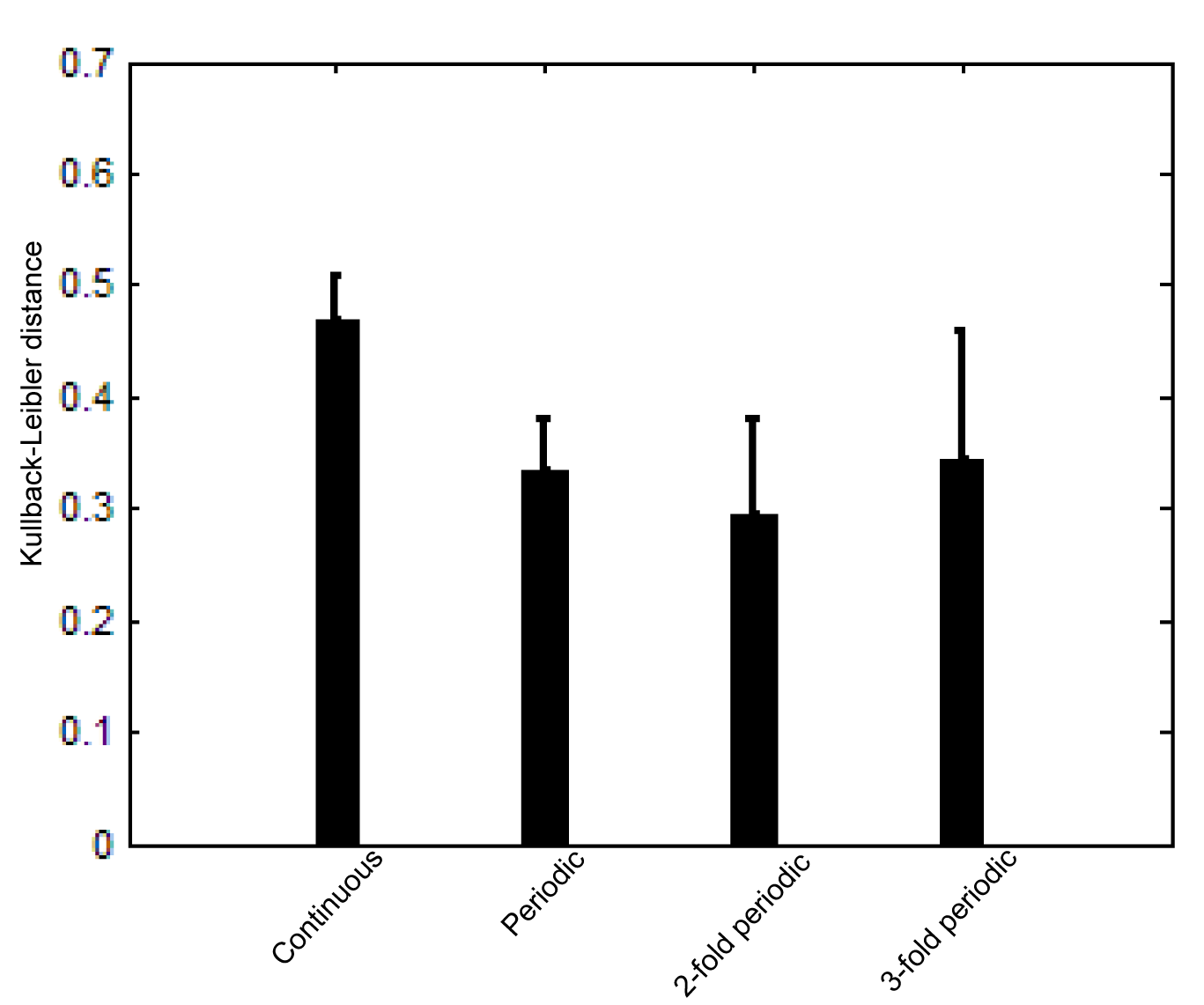

Supplement: Figure S9 — KL distances obtained under the assumption that the somite length corresponds to seven notochordal cells. The KL distances for the four types of simulation: the continuous (z = 1), periodic (z = 7), two-fold periodic (z = 14) and three-fold periodic (z = 21) models. For each model, the KL distance was calculated 300 times, and the mean and standard deviation were determined. (TIF) [file pcbi.1003957.s009.tif]
